# Effects of three frequencies of self-monitored blood glucose on HbA1c and quality of life in patients with type 2 diabetes with once daily insulin and stable control: a randomized trial

**DOI:** 10.1186/s13104-018-3138-7

**Published:** 2018-01-15

**Authors:** Johanna Hortensius, Nanne Kleefstra, Gijs W. D. Landman, Bas T. Houweling, Klaas H. Groenier, Jaap J. van der Bijl, Henk Bilo

**Affiliations:** 1 Langerhans Medical Research Group, Ommen, The Netherlands; 2 Isala Hospital, Zwolle, The Netherlands; 3 Department of Internal Medicine, University Medical Center Groningen and University of Groningen, Groningen, The Netherlands; 4 Department of Internal Medicine, Gelre Hospitals Apeldoorn, Albert Schweitzerlaan 31, 7334 DZ Apeldoorn, The Netherlands; 5 Department of Epidemiology, University Medical Center Groningen and University of Groningen, Groningen, The Netherlands; 6 Department of General Practice, University Medical Center Groningen and University of Groningen, Groningen, The Netherlands; 7 Faculty of Health, Welfare and Sports, Inholland University of Applied Sciences, Amsterdam, The Netherlands; 8 Stichting Onderzoekscentrum Ketenzorg Chronische Ziekten, Zwolle, The Netherlands

**Keywords:** Frequency, Self-monitoring of blood glucose, Type 2 diabetes, Insulin-treated

## Abstract

**Objective:**

The optimal frequency of self-monitoring of blood glucose (SMBG) in patients with type 2 diabetes (T2DM) with stable glycemic control is unknown. This study investigated effects of 3 frequencies of SMBG on glycemic control and quality of life after 9 months in patients using one long-acting insulin injection a day. In an open-label, multi-center, primary-care, parallel (1:1:1) randomized trial in the Netherlands including patients with T2DM, HbA1c ≤ 58 mmol/mol (≤ 7.5%), stable glycemic control, treated with one insulin injection daily, three frequencies of 4-point glucose measurements (before meals and bedtime) were weekly (n = 22), every 2 weeks (n = 16) and monthly (n = 20) were compared.

**Results:**

A total of 58 patients with T2DM were included by 38 general practitioners, which was lower then anticipated. There were no significant between group differences in HbA1c (mmol/mol); group C compared to A and B; − 2.7 (95% CI − 6.4, 1.0) and − 1.0 (95% CI − 4.9, 3.0) and quality of life. Baring in mind the lower than anticipated inclusion rate, there were no significant differences in HbA1c and quality of life between three different frequencies of SMBG in patients with stable glycemic control using one long-acting insulin injection.

*Trial registration* NCT01460459, registered 10-2011, recruitment between 05-2011 and 12-2011

**Electronic supplementary material:**

The online version of this article (10.1186/s13104-018-3138-7) contains supplementary material, which is available to authorized users.

## Introduction

Self-monitoring of blood glucose (SMBG) is used to improve safety, through early detection of hypoglycemia, and efficacy of insulin use [1–3]. SMBG is mostly studied in patients who have worsening glycemic control and unnecessary intense SMBG in patients with a stable glycemic control could negatively affect quality of life [3–5].

There are no studies comparing effects of different SMBG frequencies on both glycemic control and quality of life in patients with T2DM treated with one insulin injection daily or a stable glycemic control. In the Netherlands, 4-point (before meals and before bedtime) SMBG is advices once every 1–2 weeks in patients on insulin, although there are relevant differences in advices given by individual healthcare providers [6]. The largest between health-care provider differences in SMBG frequency recommendations concern those using basal insulin [6–9].

The aim of the study was to investigate effects of three different frequencies of 4-point SMBG on glycemic control and quality of life in patients with T2DM with stable glycemic control using one long-acting insulin injection daily. The study design aimed reflect the real life primary care setting in which SMBG is applied in the Netherlands.

## Main text

### Methods

#### Design

A multi-centre, open label, randomized, parallel group design. Three different (1:1:1) frequencies of 4-point SMBG (before meals and bedtime) were compared during a 9-month intervention period. The study was carried out between March 2011 and October 2012. The study and the informed consent procedure were approved by the local medical ethics committee of the Isala Hospital, Zwolle, The Netherlands. All patients gave written informed consent.

#### Study sample

From 341 general practitioners (GPs) invited, 68 GP’s agreed to participate and between 1–3 patients of 38 different GPs were included. Eligibility criteria were; adult patients, T2DM, using one long-acting insulin injection daily, performing SMBG for at least 1 year, with stable glycemic control defined as a HbA1c ≤ 58 mmol/mol the preceding 12 months and with sufficient knowledge of the Dutch language. Exclusion criteria were hypoglycemia-unawareness and serious co-morbidity evaluated by the treating GPs.

#### Study groups and procedure

Patients were randomly assigned to one of the three groups at the first study visit with block randomization, using blocks of 17 and 16, with sealed non-transparent envelopes, containing letters A, B or C. Patients were instructed to perform a 4-point SMBG; before meals and bedtime. Group A, B and C performed 4-point SMBG every week, every 2 weeks and every month, respectively.

Using validated blood glucose monitors [8] patients were instructed to record in their study diary, all additional SMBG measurements and the reason for taking extra measurements. Extra glucose measurements in patients with worsening glycemic control were allowed, patients and health care providers were advised to return to the allocated SMBG frequency as soon as was possible.

End-points were collected during routine visits and all patients received usual care offered by their healthcare providers. In the Netherlands routine visits, including HbA1c, take place every 3 months. No financial compensation was provided.

#### Outcome measures

The primary end-point was the between group difference in HbA1c (mmol/mol). A difference of 5.5 mmol/mol (> 0.5%) was regarded as clinically relevant [10, 11]. Secondary endpoints were health-related quality of life and diabetes self-care. Other endpoints were the number of recorded hypo- and hyperglycemic events and the fasting blood glucose concentrations.

#### Measurements

Data collected at baseline included: diabetes duration, duration of SMBG, medication, blood pressure, length, weight, smoking status, alcohol status and micro- and macrovascular complications (yes or no). Data on additional diabetes-related contacts with the healthcare provider and changes in diabetes medication were recorded in the study diaries.

At baseline and 9 months, three validated questionnaires were completed. The 12-item Short Form Health Survey (SF-12) [12, 13], the Problem Areas in Diabetes (PAID) [14]. Higher scores indicate higher emotional distress. The Summary of Diabetes Self Care Activities (SDSCA) [15], see Additional file 1 for more information on the questionnaires.

#### Sample size calculation

This was calculated using software PASS-2008. To detect a relevant difference in HbA1c of 5.5 mmol/mol (0.5%) [10, 11], a SD of 6.8 mmol/L, with a power of 90%, an alpha of 0.05/3, 2-tailed, the sample size of the study was 129, with 43 patients in each group. The SD was derived from the prospective ZODIAC cohort [16], no randomized trials with a comparable research question were available.

#### Statistical analysis

Data entry was performed in duplicate and statistical analyses were carried out by a statistician blinded for treatment allocation. Study end-points were analyzed using a mixed model. Fixed factors were study arm and total number of SGBM; patients were random factors and baseline HbA1c was a covariate. The fasting blood glucose concentrations were used to investigate if there were differences between the blood glucose concentrations, with baseline fasting blood glucose concentrations as covariates. Bonferroni correction was used to adjust for multiple testing. The response variables smoking and alcohol use were coded into binary variables and analyzed using Generalized Linear Models (GLM), with a binary distribution and logit link function.

Analyses were performed according to the intention-to-treat principle. A predefined per-protocol analysis was performed for the primary end-point only in patients who were compliant with SMBG frequency (who performed 80% or more of the required glucose measurement). p values were tested two-tailed.

### Results

#### Patients

**Fig. 1:**
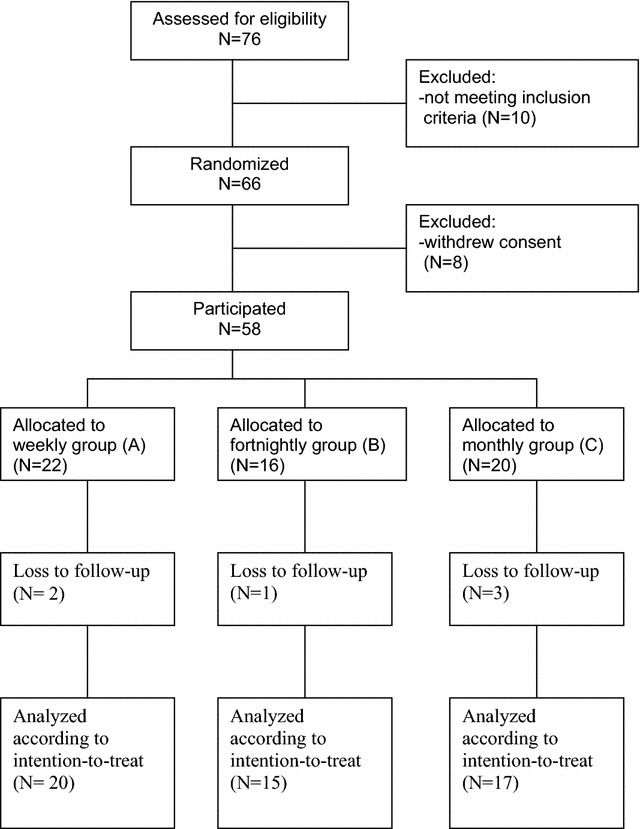
Flow diagram. Number of participants in stages of the trial

Sixty-six patients were randomized, 8 withdrew consent shortly after randomization before completion of baseline measurements and 58 patients participated. Six patients were lost for follow-up and 52 were randomised. See Fig. 1 that shows the flow of patients throughout the trial.

The study stopped prematurely due to the slow anticipated inclusion rate during the study period. Reasons provided for the slow recruitment rate were primarily difficulty of finding patients with a stable glycemic control who mostly were used to specific SMBG frequency for a long time, who wanted to change SMBG frequency for the duration of the study. Other arguments were lack of time, participation in other trials and lack of financial compensation by care providers.

The baseline characteristics are presented in Table 1. A total of 65% of the patients group A, 63% of the patients in group B and 59% of the patients in group C performed at least 80% of the requested glucose measurements. All patients reported to be compliant with their insulin injection. See Additional file 1 on changes in insulin dose.

**Table 1:** Baseline characteristics

	A: SMBG 1 day weekly (n = 22)	B: SMBG 1 day every 2 weeks (n = 16)	C: SMBG 1 day monthly (n = 20)
Gender (male)	13 (59)	8 (50)	12 (60)
Age (years)	67 ± 12	69 ± 9	65 ± 9
Diabetes duration (year)	11 (6, 15)	8 (6, 13)	13 (7, 17)
Duration SMBG (year)	4 (2, 7)	4 (3, 7)	5 (4, 6)
HbA1c (mmol/mol)	51 ± 4.7	52 ± 4.6	51 ± 5.6
Fasting blood glucose (mmol/L)	6.7 (6.0, 7.2)	5.9 (4.8, 6.4)	6.5 (5.4, 8.2)
Dose of insulin (units)	22 (16)	30 (22)	20 (15)
Metformin	21 (95%)	14 (88%)	16 (80%)
Tolbutamide	4 (18%)	4 (25%)	7 (35%)
Gliclazide	5 (23%)	4 (25%)	3 (15%)
Glimepiride	3 (14%)	2 (13%)	3 (15%)
Body mass index (kg/m^2^)	30 ± 4.1	30 ± 2.8	30 ± 3.6
Systolic blood pressure (mmHg)	141 ± 14	134 ± 11	133 ± 16
Cholesterol/HDL ratio	3.30 (2.9, 4.0)	3.60 (3.3, 4.3)	3.40 (2.8, 4.4)
Macrovascular complication (yes)	5 (23)	6 (38)	5 (25)
Serum creatinine (μmol/L)	71 (63)	73 (60, 97)	76 (63, 82)
Albumin creatinine ratio	1.0 (0.3, 2.0)	1.6 (0.99, 4.8)	0.8 (0.5, 2.3)
Retinopathy (yes)	0 (0)	1 (6)	3 (15)
Dose of insulin (units)	22 (16, 29)	30 (22, 36)	20 (15, 39)
Smoking (yes)	4 (18)	2 (13)	3 (15)
Alcohol use (yes)	12 (55)	6 (38)	10 (50)

Data are mean ± SD, n (% of known data) or median (P25, P75)

Table 2 shows HbA1c levels at different time points and the estimated changes from baseline to 9 months. There were no significant changes within and between groups. Subgroup of patients who were compliant with the allocated SMBG frequency and analysis in fasting capillary glucose concentrations, see Additional file 1.

**Table 2:** HbA1c levels (mmol/mol) at different time points and estimated changes between and within groups

SMBG frequency	Baseline	3 months	6 months	9 months	Estimated changes within groups^a^
Weekly (A)	51.1 ± 4.7	52.1 ± 5.4	52.2 ± 5.0	54.4 ± 6.8	3.32 (− 1.38, 8.01)
Every 2 weeks (B)	51.6 ± 4.6	51.3 ± 7.8	50.2 ± 5.8	53.1 ± 5.8	1.50 (− 4.01, 7.01)
Monthly (C)	51.2 ± 5.6	51.1 ± 5.6	50.2 ± 9.0	51.8 ± 6.6	0.60 (− 4.33, 5.53)

Time (months)	Groups^b^		
	B minus A	C minus A	C minus B
3	− 0.62 (− 4.83, 3.59)	− 1.16 (− 5.10, 2.79)	− 0.53 (− 4.83, 3.77)
6	− 2.40 (− 7.31, 2.51)	− 2.51 (− 7.12, 2.10)	− 0.11 (− 5.02, 4.80)
9	− 1.72 (− 5.60, 2.17)	− 2.70 (− 6.35, 0.95)	− 0.98 (− 4.94, 2.98)

Data are mean ± SD, estimated changes (95% CI, Bonferroni corrected). All p values > 0.05

^a^From baseline to 9 months

^b^Estimated changes between groups adjusted for baseline differences

The changes in the outcome of the SF-12, PAID and SDSCA are presented in Additional file 1. There were significant changes between the groups in only two dimensions of the SF-12, the subscale role limitation (emotional problems) and vitality. In improvement in vitality was most pronounced the lowest SMBG frequency.

#### Safety

Eight patients reported at least one mild hypoglycemic event and consequently performed extra SMBG measurements; 2 patients in group A, 3 in group B and 3 in group C. No glucose values below three were reported. No symptoms of hypoglycemia were reported.

### Discussion

Three intensities of performing 4-point SMBG; weekly, every 2 weeks, every month, were compared in patients with T2DM, who used one long-acting insulin injection daily and had a stable glycemic control for at least 12 months. There were no significant differences in glycemic control, quality of life, diabetes self care activities, fasting blood glucose or hypoglycemia events.

This is the first study that evaluated effects of 3 different SMBG frequencies on glycemic control and quality of life in patients with T2DM with stable control. SMBG is used for monitoring occurrence of possible hypoglycemia and improving glycemic controls [17]. Several studies indicated that SMBG potentially also has an impact on quality of life [3]. Whether more intensive SMBG negatively affects quality of life in patients with a stable glycemic control is unknown. The evidence for efficacy of SMBG is somewhat conflicting concerning HbA1c [2, 18–20]. Some reported no significant relationships between SMBG and HbA1c [21, 22]. While in other studies, results indicated that SMBG resulted in improvements in HbA1c in the order of 3.5–7.0 mmol/mol. Most previous studies were either not randomized, included patients with unstable glycemic control or patients using more intensive insulin regimens [2, 18–20].

Although this is the first study to investigate both SMBG frequency and QoL, it was unfortunate that there was a lower then anticipated inclusion rate. There were a variety of reasons for the slow recruitment rate. The most important reason reported by GPs was that those with a stable glycemic control were used to specific SMBG frequencies and did not want to change SMBG frequency for the duration of the study.

### Conclusion

This is the first study investigating effects of SMBG in stable patients, in this study there were no significant between-group differences in glycemic control or quality between 3 frequencies of 4-point SMBG in patients with T2DM with stable glycemic control using one insulin injection. The study was limited by a slower then anticipated inclusion rate. It was difficult to recruit patients with a stable glycemic control. Nevertheless, it could be very interesting to repeat this study, with extra focus on recruitment rate and for example financial compensation for patients, to establish whether the currently advised intensive SMBG in this patient group is warranted.

## Limitations

The major limitation of this study was the inability to recruit the pre-planned 150 patients. We were able to include 58 patients in the predefined time frame.

Another limitation was that in the absence of results from randomized trials, the assumptions for the sample size calculation were derived from patients included in the prospective ZODIAC cohort study [16]. Retrospectively, it could have been that the assumptions on which the power calculation was based were inaccurate. Patients who contributed to the sample size calculation, retrospectively, differed substantially from patients included in this study; for example the average was HbA1c of 64 mmol/mL (SD 6.8 mmol/mol). Although the upper bounds of the confidence intervals which were within the pre-specified relevancy margin of 5.5 mmol/mol (0.5%) gives some indication on the change of a type 2 error. This confidence interval could indicate that either no relevant differences were to be expected or it could indicate the presence of a type 2 error. The assumptions for the power calculation were, in the absence of comparable randomized trials, based on prospective observational cohort data and in retrospect, could even have been too conservative.

Other limitations were the relatively wide confidence intervals of the results from the analysis of the subscales of the SF-12 and the SDSCA, which could point to a lack of power. Although the results were bonferroni corrected, the significant difference in the SF-12 subscale vitality could still be the results of multiple testing. Furthermore, we did not collect information on SMBG habits prior to randomization nor checked whether medication use collected from patient dairies corresponded to actual medication compliance. Compliance was checked by self-reports using study diaries not on glucose readings from patients glucose monitors. The cut-off for hypoglycemia a priori was set at 3.5 mmol/L, we acknowledge that there is debate on the optimal cut-off value, therefore we added post hoc results for the cut-off value for hypoglycemia of 3.0 mmol/L [23]. We also acknowledge that the recommended SMBG frequencies vary substantially between health care providers and between countries [6, 24]. In nine patients the insulin dose was changed which effected the between group differences.

A strength was the real life setting were for example extra glucose measurements and dose changes of insulin in patients with worsening glycemic control were allowed. Other strengths were; the use of different frequencies of SMBG, based on recommendations made in the Dutch guideline currently used in daily practice [6].

## Additional file

**Additional file 1.** Questionnaire outcomes, additional methodology and results. A. Changes in questionnaires outcome, the SF-12, PAID and SDSCA, after 9 months. B. Methodology Questionnaires & Handling of changes in insulin dose. C. Additional results. Data are mean differences within and between groups (95% CI) (Bonferroni corrected) Range: scores per subscale. * Significant differences. ^±^ Higher PAID scores indicate more diabetes-related emotional distress. Subgroup analyses of patients who were compliant with the allocated SMBG frequency. The mean between group differences in fasting capillary glucose concentrations.

## References

[1] Nathan DM, McKitrick C, Larkin M, Schaffran R, Singer DE (1996). Glycemic control in diabetes mellitus: have changes in therapy made a difference?. Am J Med.

[2] Karter AJ, Ackerson LM, Darbinian JA, D’Agostino RB, Ferrara A, Liu J, Selby JV (2001). Self-monitoring of blood glucose levels and glycemic control: the Northern California Kaiser Permanente Diabetes registry. Am J Med.

[3] Hortensius J, Kars MC, Wierenga WS, Kleefstra N, Bilo HJ, van der Bijl JJ (2012). Perspectives of patients with type 1 or insulin-treated type 2 diabetes on self-monitoring of blood glucose: a qualitative study. BMC Public Health.

[4] Vincze G, Barner JC, Lopez D (2004). Factors associated with adherence to self-monitoring of blood glucose among persons with diabetes. Diab Educ.

[5] Wa F (2007). Barriers and behaviours in blood glucose monitoring. US Endocr Dis..

[6] Hortensius J, Kleefstra N, Houweling ST, van der Bijl JJ, Gans RO, Bilo HJ (2012). What do professionals recommend regarding the frequency of self-monitoring of blood glucose?. Neth J Med.

[7] Federation DD. Multidisciplinary guideline self-monitoring of blood glucose by people with diabetes. 2012. http://www.eadv.nl/page/Richtlijnen-1/Zelfcontrole-open?mod%5B1238%5D%5Bi%5D=57. Accessed 1 Dec 2013.

[8] Berard LD, Blumer I, Houlden R, Miller D, Woo V (2013). Monitoring glycemic control. Can J Diab.

[9] A. American Diabetes (2013). Standards of medical care in diabetes—2013. Diabetes Care.

[10] Kleefstra N, Hortensius J, Logtenberg SJ, Slingerland RJ, Groenier KH, Houweling ST, Gans RO, van Ballegooie E, Bilo HJ (2010). Self-monitoring of blood glucose in tablet-treated type 2 diabetic patients (ZODIAC). Neth J Med.

[11] Kleefstra N, Hortensius J, van Hateren KJ, Logtenberg SJ, Houweling ST, Gans RO, Bilo HJ (2009). Self-monitoring of blood glucose in noninsulin-treated type 2 diabetes: an overview. Diab Metabol Syndr Obes Targets Ther.

[12] Gandek B, Ware JE, Aaronson NK, Apolone G, Bjorner JB, Brazier JE, Bullinger M, Kaasa S, Leplege A, Prieto L, Sullivan M (1998). Cross-validation of item selection and scoring for the SF-12 Health Survey in nine countries: results from the IQOLA Project. International Quality of Life Assessment. J Clin Epidemiol.

[13] Keller SD, Majkut TC, Kosinski M, Ware JE (1999). Monitoring health outcomes among patients with arthritis using the SF-36 Health Survey: overview. Med Care.

[14] Snoek FJ, Pouwer F, Welch GW, Polonsky WH (2000). Diabetes-related emotional distress in Dutch and U.S. diabetic patients: cross-cultural validity of the problem areas in diabetes scale. Diabetes Care.

[15] Toobert DJ, Hampson SE, Glasgow RE (2000). The summary of diabetes self-care activities measure: results from 7 studies and a revised scale. Diabetes Care.

[16] Ubink-Veltmaat LJ, Bilo HJ, Groenier KH, Houweling ST, Rischen RO, Meyboom-de Jong B (2003). Prevalence, incidence and mortality of type 2 diabetes mellitus revisited: a prospective population-based study in The Netherlands (ZODIAC-1). Eur J Epidemiol.

[17] World Health Organization (2003). Adherence to long-term therapies. Evidence for action.

[18] Murata GH, Shah JH, Hoffman RM, Wendel CS, Adam KD, Solvas PA, Bokhari SU, Duckworth WC, S. Diabetes Outcomes in Veterans (2003). Intensified blood glucose monitoring improves glycemic control in stable, insulin-treated veterans with type 2 diabetes: the Diabetes Outcomes in Veterans Study (DOVES). Diabetes Care.

[19] Schutt M, Kern W, Krause U, Busch P, Dapp A, Grziwotz R, Mayer I, Rosenbauer J, Wagner C, Zimmermann A, Kerner W, Holl RW, DPV Initiative (2006). Is the frequency of self-monitoring of blood glucose related to long-term metabolic control? Multicenter analysis including 24,500 patients from 191 centers in Germany and Austria. Exp Clin Endocrinol Diab..

[20] Secnik K, Yurgin N, Lage MJ, McDonald-Everett C (2007). Patterns of blood glucose monitoring in relation to glycemic control among patients with type 2 diabetes in the UK. J Diabetes Complications.

[21] Evans JM, Newton RW, Ruta DA, MacDonald TM, Stevenson RJ, Morris AD (1999). Frequency of blood glucose monitoring in relation to glycaemic control: observational study with diabetes database. BMJ.

[22] Harris MI, National H, S. Nutrition Examination (2001). Frequency of blood glucose monitoring in relation to glycemic control in patients with type 2 diabetes. Diabetes Care.

[23] G. International Hypoglycaemia Study (2017). Glucose concentrations of less than 3.0 mmol/L (54 mg/dL) should be reported in clinical trials: a joint position statement of the American Diabetes Association and the European Association for the Study of Diabetes. Diabetes Care.

[24] Hortensius J, van der Bijl JJ, Kleefstra N, Houweling ST, Bilo HJ (2012). Self-monitoring of blood glucose: professional advice and daily practice of patients with diabetes. Diab Educ.

