# Supplementary material for: Effects of three frequencies of self-monitored blood glucose on HbA1c and quality of life in patients with type 2 diabetes with once daily insulin and stable control: a randomized trial
Source: BMC Res Notes. 2018 Jan 15;11:26. doi: 10.1186/s13104-018-3138-7 (PMC5769429; doi:10.1186/s13104-018-3138-7)
Supplement: Supplementary file 1 — Additional file 1. Questionnaire outcomes, additional methodology and results. A. Changes in questionnaires outcome, the SF-12, PAID and SDSCA, after 9 months. B. Methodology Questionnaires & Handling of changes in insulin dose. C. Additional results. Data are mean differences within and between groups (95% CI) (Bonferroni corrected) Range: scores per subscale. * Significant differences. ± Higher PAID scores indicate more diabetes-related emotional distress. Subgroup analyses of patients who were compliant with the allocated SMBG frequency. The mean between group differences in fasting capillary glucose concentrations. [file 13104_2018_3138_MOESM1_ESM.docx]

Additional file: between group differences in questionnaires.

|  | **Changes within weekly group (A)** | **Changes within fortnightly group (B)** | **Changes within monthly group (C)** | **Changes between group B vs A (95%CI)** | **Changes between group C vs A (95%CI)** | **Changes between group C vs B (95%CI)** |
| --- | --- | --- | --- | --- | --- | --- |
| **SF-12 scale** |  |  |  |  |  |  |
| Physical function  Range: 2 - 6 | -0.03  (-0.43,0.38) | -0.38  (-0.83,0.10) | -0.06  (-0.48,0.37) | -0.19  (-0.57,0.19) | 0.19  (-0.17,0.55) | 0.38  (-0.01,0.76) |
| Social Function  Range: 1 – 5 | 0.27  (-0.37,0.91) | -0.33  (-1.07,0.40) | 0.17  (-0.50,0.84) | -0.40  (-1.00,0.20) | 0.13  (-0.44,0.70) | 0.53  (-0.08,1.14) |
| Role limitations (physical problem)  Range: 2 - 4 | -0.08  (-0.34,0.19) | -0.07  (-0.37,0.24) | 0.17  (-0.11,0.44) | -0.01  (-0.26,0.23) | 0.22  (-0.01,0.46) | 0.23  (-0.02,0.49) |
| Role limitation (emotional problem)  Range: 2 - 4 | 0.13  (-0.08,0.33) | 0.03  (-0.20,0.27) | 0.08  (-0.13,0.30) | 0.01  (-0.18,0.21) | **0.19**  **(0.01,0.38)***  **P=0.04** | 0.18  (-0.02,0,38) |
| Mental health:  Range: 2 – 12 | 0.18  (-0.37,0.73) | -0.23  (-0.86,0.39) | 0.06  (-0.51,0.63) | -0.21  (-0.72,0.30) | 0.15  (-0.34,0.63) | 0.36  (-0.16,0.88) |
| Vitality  Range: 1 - 6 | -0.23  (-0.95,0.50) | -0.47  (-1.29,0.36) | 0.22  (-0.53,0.98) | -**0.89**  **(-1.56,-0.21)***  **P=0.01** | 0.14  (-0.51,0.78) | **1.02**  **(0.34,1.71)***  **P=0.001** |
| Bodily pain  Range: 1 – 5 | 0.41  (-0.24,1.06) | -0.35  (-1.12,0.41) | 0.28  (-0.40,0.96) | 0.02  (-0.60,0.64) | 0.30  (-0.28,0.88) | 0.28  (-0.35,0.91) |
| General health  Range: 1 – 5 | -0.10  (-0.61,0.41) | -0.13  (-0.72,0.46) | -0.17  (-0.70,0.37) | -0.13  (-0.61,0.34) | 0.03  (-0.43,0.48) | 0.16  (-0.33,0.65) |
| **PAID^±^**  Range: 0- 4 | 0.01  (-0.36,0.37) | 0.27  (-0.16,0.69) | 0.06  (-0.33,0.45) | 0.06  (-0.29,0.40) | 0.10  (-0.23,0.43) | 0.05  (-0.31,0.40) |
| **SDSCA** |  |  |  |  |  |  |
| General diet  Range: 0 – 7 | 0.78  (-0.23,1.78) | -0.30  (-1.47,0.87) | -0.06  (-1.12,1.01) | 0.10  (-0.85,1.04) | -0.50  (-1.40,0.40) | -0.60  (-1.59,0.37) |
| Specific diet  Range: 0 – 7 | -0.30  (-0.97,0.37) | 0.06  (-0.72,0.84) | 0.14  (-0.56,0.84) | 0.27  (-0.36,0.90) | -0.22  (-0.82,0.37) | -0.50  (-1.14,0.15) |
| Foot care  Range: 0 – 7 | 0.10  (-1.30,1,50) | 0.30  (-1.32,1.92) | -0.47  (-1.95,1.01) | -0.09  (-1.41,1.22) | 0.05  (-1.20,1.30) | 0.14  (-1.20,1.49) |
|  | -0.60  (-2.01,0.81) | -0.87  (-2.50,0.77) | 0.33  (-1.16,1.82) | -0.35  (-1.67,0.97) | -0.07  (-1.33,1.19) | 0.28  (-1.08,1.63) |

**Methodology**

*Questionnaires*

The 12-item Short Form Health Survey (SF-12) consists of eight subscales and are a selection from the SF-36 [12, 13]. Higher scores indicate better general quality of life. The Problem Areas in Diabetes (PAID) is a 20-item scale to assess the current level of diabetes-related emotional distress[14]. Higher scores indicate higher emotional distress. The Summary of Diabetes Self Care Activities (SDSCA )assesses the self-care activities during the past seven days, using a 13 item scale[15]. A total of 11 items represent the number of days on which activities were performed in the last week, two items concern smoking status and use of alcohol.

*Changes in insulin dose*

The insulin dose was changed at least once time in six patients in group A, two in group B and eight in group C. Doses of oral glucose lowering agents were changed in seven patients in group A (32%), six patients in group B (38%) and nine group C (45%). Additional diabetes-related contacts were reported for two patients: one patient in group B and one in group C.

**Results:**

*Subgroup analyses of patients who were compliant with the allocated SMBG frequency*

The mean differences were, group B compared to group A: -1.20 mmol/mol (95%CI: -5.63 - 3.24), group C compared to group A: -4.41 mmol/mol (95%CI: -8.90 - 0.09) and group C compared to group B: -3.21 mmol/mol (95%CI: -8.02 - 1.60).

*The mean differences in fasting capillary glucose concentrations*

Group B compared to group A; -0.38 mmol/L (95%CI: -0.80 - 0.04), group C compared to group A: -0.23 mmol/L (95%CI: -0.64 - 0.19) and group C compared to group B: 0.15 mmol/L (95%CI: -0.32 - 0.62).
